# Diagnosis routine and approach in genetic sensorineural hearing loss

**DOI:** 10.1016/S1808-8694(15)30087-2

**Published:** 2015-10-19

**Authors:** Fatima Regina Abreu Alves, Fernando de Andrade Quintanilha Ribeiro

**Affiliations:** a PhD student in Otorhinolaryngology - FCMSC -SP; M.S. in Otorhinolaryngology - FCMSC - SP, Preceptor of ENT - HSPM - SP; b PhD in Otorhinolaryngology -UNIFESP - EPM, Head Physician of the Santa Casa de Misericórdia de São Paulo. School of Medical Sciences - Santa Casa de São Paulo

**Keywords:** diagnosis, genetics, hearing loss, sensorineural

## Abstract

A**im**: To develop a screening in order to determine the more common syndromic and non-syndromic genetic SNHL, considering epidemiological data, information and the development of new technologies; clinical implications and bioethical issues. **Materials and Methods:** We reviewed the literature in order to develop a screening that includes: history, patterns of inheritance, physical evaluation, laboratory tests, image studies, multidisciplinary approaches and genetic tests. **Conclusion:** The epidemiologic data estimates that at least 50% of prelingual HL can be determined by genetic alterations. Medical and family histories are extremely important to help one achieve a genetic-based SNHL diagnosis, and help determine inheritance patterns. Through a high suspicion index, syndromic cases can be diagnosed or excluded, with a careful evaluation and molecular basis tests used to better determine the hearing loss. Genetic tests and mitochondrial inheritance should be considered in any family with many affected individuals, except when the hearing loss was clearly transmitted by a male. In cases of non-syndromic SNHL, GJB2 mutation analysis must be proposed.

## INTRODUCTION

Hearing loss (HL) is the most common sensorial disorder1, and Sensorineural Hearing Loss (SNHL) affects approximately 1 to 3 for 1000 newborn babies^2^. It is estimated that at least 50% of pre-speech stage hearing loss are caused by genetic alterations3; however, we lack accurate epidemiological data about post-speech hereditary hearing loss^1^.

**Figure 1:**
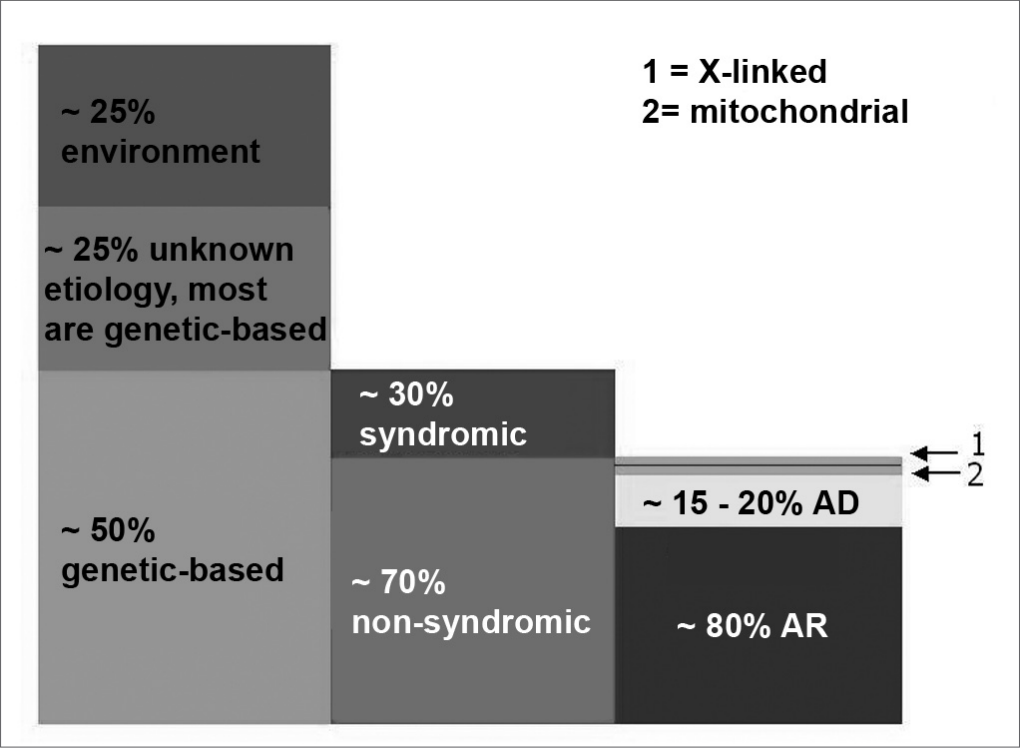
Pre-speech SNHL: epidemiological data from developed countries - this breaking down of hearing loss based on cause (column 1), presence or absence of associated traces in cases of genetic etiology (column 2) and the inheritance mode in the non-syndromic group (column 3). In column 3, box 1 represents X-linked hearing loss (approximately 1% of the non-syndromic) and box 2 represents mitochondrial hearing loss, which explains at least 1%. AR: recessive autosomal; AD: dominant autosomal. (Modified from Schrijver, 2004)

Hereditary cases are further broken down into syndromic and non-syndromic. Of pre-speech hearing losses, 70% are non-syndromic and the remaining 30% are characterized by the presence of other signs and symptoms (syndromic)1. Among non-syndromic hearing losses, 80% are recessive autosomal (DFNB), 15 to 20% are dominant autosomal (DFNA) and less than 2% are X-linked (DFN) or mitochondria linked^2^, ^3^ (Figure 1).

The syndromic hearing loss is characterized by additional manifestations such as: retinitis pigmentosa (Usher’s Syndrome), euthyroidism goiter and inner ear malformations (Pendred’s Syndrome), renal anomalies (Alport’s Syndrome) and presence of enlarged QT interval (Jervell’s and Lange-Nielsen’s Syndromes). Associated signs and symptoms are useful when they can be observed; however they can appear later on, or not be recognized at all, thus making the diagnosis incomplete^4^.

## OBJECTIVE

We propose a road map to investigate the most common genetic SNHL, considering epidemiological data, information and the development of new technologies, clinical implications and bioethical issues.

## MATERIALS AND METHODS

We carried out a careful revision, using the following keywords: hearing loss, sensorineural hearing loss, genetics and diagnosis, in order to draw the investigation and approach road map.

## LITERATURE REVIEW

**Figure 2:**
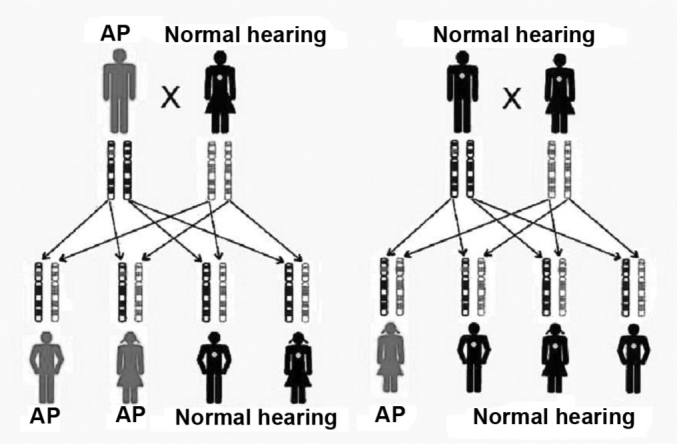
Inheritance mode - the first key represents the inheritance of a dominant autosomal mutation; a red band tells us of a mutation in the father’s gene. In the second key we see the inheritance of a recessive autosomal mutation; the red band represents a recessive mutation in a father’s gene and in the same gene belonging to the mother; in the dominant form, only one copy is necessary for the individual to be affected, and in the recessive form, both copies of the same gene must be altered. (Modified from REHM, 2003)

**Figure 3:**
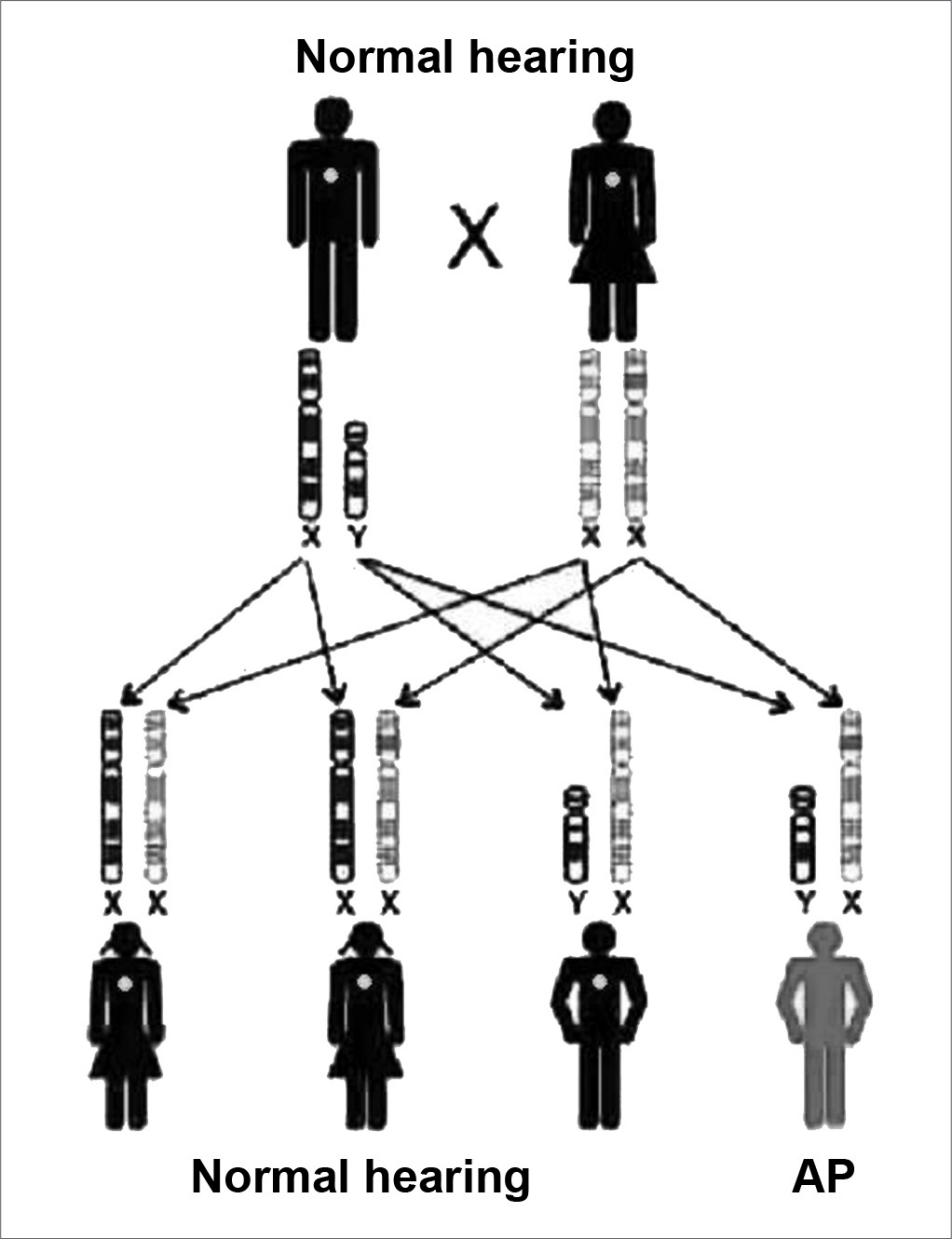
Inheritance mode - this key represents an X-linked recessive mutation; the daughters that inherit the altered copy from the mother will not be affected, because they receive one normal copy from the father, and the sons have a 50% chance of inheriting the altered X chromosome from the mother. (Modified from Rehm, 2003)

Genetic SNHL may follow a dominant autosomal pattern, recessive autosomal, X-linked or mitochondrialinked (Figures 2 and 3). The genetic basis is highly complex. Allelic mutations in some genes can cause recessive and dominant HL, mutations in this gene can cause syndromic and non-syndromic HL and, recessive HL can be caused by a combination of two mutations in different genes of the same functional group^3^, ^5^.

### Non-syndromic HL

One single gene, GJB2 (Gap Junction β2), explains more than 50% of recessive hearing loss cases. Connexin 26 (Cx26), a protein coded by GJB^2^, belongs to a family of gap-junction proteins which are responsible for the transport of ions, metabolites and secondary messengers^2^. Animal studies suggest that the Cx26 participates in the recycling of potassium ions back to the cochlear duct endolymph, after stimulation by sensorial hair cells2. This non-syndromic recessive autosomal hearing loss in the DFNB1 locus of chromosome 13q^11^, ^12^, is characterized for being congenital, typically non-progressive and moderate to profound^3^. The locus has two genes, GJB2 e GJB^63^,^6^, ^7^.

-The POU3F4 codifies a transcription factor. The HL is X-linked, non-syndromic, progressive and profound (DFN3) and may have a conductive component due to footplate fixation. CT scan can be useful; an enlargement in the internal acoustic meatus or dilation between the internal acoustic meatus and the inner ear may be seen. The perilymphatic pressure is increased and the inner ear perilymph may pour during the surgical removal of the stapes^3^.

### Syndromic HLs

Pendred’s syndrome: is responsible for 4 to 10% of the hereditary pre-speech hearing loss in the world^3^, ^4^. One of the most common forms of syndromic HL is a recessive autosomal disorder made up of HL and a defect in the organification of the thyroid hormone, thus forming an euthyroidism goiter. The goiter is not consistently present and sometimes is manifested only in adults3. An enlargement in the vestibular aqueduct was found in almost all the patients and is associated with a dysmorphic cochlea, which has 1.5 and not 2.5 turns (Mondini’s dysplasia)3,4. The HL is characteristically pre-speech (not necessarily congenital), sensorineural or rarely mixed, from severe to profound, frequently stable; however it may be fluctuating and progressive^3^, ^4^. Mutations in the SLC26A4 gene, also known as PDS, respond for the majority, if not for all the cases of Pendred’s syndrome. A useful laboratorial test for the diagnosis is the test of perchlorate discharge, and we may also use CT scan^38^,^9^.

Usher’s syndrome: recessive autosomal disorder, characterized by HL, progressive eyesight loss because of retinitis pigmentaris and, in some cases, balance disorders. The syndrome is genetically heterogeneous. There are different genes that may cause the syndrome. It is clinically divided in: type 1, type 2, type 3 and atypical. The type 1 Usher’s syndrome is characterized by severe to profound congenital HL, retinitis pigmentaris that starts in the pre-pubertal age, and the lack of vestibular reflexes. HL in Usher’s syndrome types 1 and 2 is congenital, while retinitis pigmentaris may be of late onset and not noticed until adolescence4. Estimated prevalence is of 3 to 4.5 in 100,00010. The VIIa myosin gene is responsible for type 1B Usher’s syndrome; the VIIa myosin is expressed in the hair cells of the Organ of Corti and in the vestibule; in the retina, the VIIa myosin is present in the retina pigmented epithelial cells^10^.

Alport’s syndrome: caused by alterations in the type IV collagen chains; and symptoms reflect the basal membrane involvement in different organs. The X-linked inheritance is predominant in 85% of the cases, and the recessive autosomal form is responsible for 15% of the cases. It is characterized by hematuria, which evolves to renal failure and may be accompanied by SNHL and ocular defects. The incidence of Alport’s syndrome is reported as being 1 in 200 thousand. The HL is a frequent finding and one of the first symptoms of this syndrome, being also a relevant prognostic factor in regards of the renal involvement. The HL is of variable intensity, progressive, bilateral and symmetrical, involving the middle range and high frequencies. In investigating SNHL in children with hematuria, in adolescents and male adults in end-stage renal failure, and in patients with family history of renal disease in siblings or relatives in their mothers’ side, one must consider the diagnosis of Alport’s syndrome. Because of its complexity and high costs, the available genetic diagnostic tests are restricted to selected cases^11^, ^12^, ^13^.

Jervell’s and Lange-Nielsen’s syndrome: mutations in KCNQ1 or KCNE1, recessive autosomal inheritance, delayed repolarization of the potassium channel, with profound SNHL, cochleo-saccular dysplasia (Scheibe), abnormal cardiac conduction, prolonged QT interval and sudden death. The cardiac problem may pass undiagnosed and it may prove difficult to detect^1^, ^4^, ^14^.

### Mitochondrial HLs

SNHL is present in 42 to 70% of the individuals with mitochondrial disorders and may be syndromic or not. Mutations in the mitochondrial DNA were identified in approximately 3% of the patients with SNHL, and the mutations are transmitted exclusively from the mother^3^. Among the patients who receive conventional treatment with aminoglycosides (at therapeutic levels and for a short period of time), more than 25% presented SNHL; and 50% of them are carriers of the 12S rRNA mutation. The genetic mitochondrial HL is based on the high need of ATP by the cochlear hair cells, and the reduction in available ATP - caused by mitochondrial oxidative phosphorylation dysfunction brought about by the mutations, results in disorders of the ionic gradient in the inner ear. Mitochondrial mutations may be related to age-related progressive hearing loss - presbycusis^3^.

Mitochondrial SNHL may be syndromic. Seen in the Kearns-Sayre syndrome (progressive ophthalmoplegia and HL); in the mitochondrial encephalopathy with lactic acidosis and stroke episodes (MELAS) or, in diabetes and maternal inheritance HL^3^.

### Genetic Hearing Loss Assessment

As soon as one suspects of genetic SNHL, a complete pre-natal, medical and family history should be collected. The clinical and genetic exams are necessary in order to rule out characteristics which are common to the syndromic or congenital infectious etiology. An ophthalmology exam must be carried out, since ocular alterations are present in more than half the children with HL from severe to profound. Lab tests must be individualized and directed according to diagnostic suspicion. TSH and perchlorate discharge test in the suspicion of Pendred’s syndrome; urine test and renal function test in children with possible Alport’s syndrome; an EKG to assess the QT interval in the Jervell’s and Lange-Nielsen’s syndromes. Image studies may include high resolution CT scans in order to assess Mondini’s malformation or an MRI to see the auditory nerve, rule out aplasia or infectious inner ear destruction, being especially important before a cochlear implant surgery^3^. Audiograms are important components of the assessment process^15^. Genetic tests have implications to all family members, and the genetic confidentiality of the patient’s relatives must be considered^15^. Family history may stigmatize family members, it may be necessary to contact family members in order to reach an accurate interpretation of results, always respecting patients’ privacy and autonomy.

The array of molecular tests clinically available or still under investigation may be obtained from the website: Genetests (http://www.genetests.org/servlet/access)^3^, ^16^, ^17^, ^18^, ^19^.

## DISCUSSION

Detailed family history is one of the most important clues as to the HL etiology, defining the inheritance pattern in the family. It is important to collect details on the general health and hearing of siblings, parents, grandparents and other close relatives. It is also relevant to check for consanguinity and family ethnic background. A careful physical exam may identify characteristics of syndromes or confirm an isolated case (non-syndromic).

As we mentioned before, the laboratorial tests must be confirmed according to clinical suspicion. The audiograms are important to determine the degree of hearing loss, in order to follow the HL in evolutional cases and also to guide patient rehabilitation. It is important to compare the patient’s audiologic results with those of other family members.

The assessment of a patient with HL requires a multidisciplinary approach and must include advice and support to the parents, and genetic counseling aims at optimizing the use of the most adequate clinical resources.

Family genetic evaluation is also paramount in the diagnostic process and that of ordering specific genetic tests in a child with hearing impairment. Parents should be informed on the HL cause and behavior, whether or not it impacts other organs and the possibility of it affecting other children in the family or family members. Genetic tests are integral part of the assessment in an attempt to confirm the specific diagnosis. Parents must be informed on the diagnosis and conditions that follow the clinical manifestation, prognosis, inheritance mode and treatment options. If the genetic tests result in important health consequences for the health of relatives, they must be contacted. Pre and post testing genetic counseling is important for the patient to understand the advantages and limitations of a particular genetic test, as well as the resulting consequences for the patient and his family.

**Figure 4:**
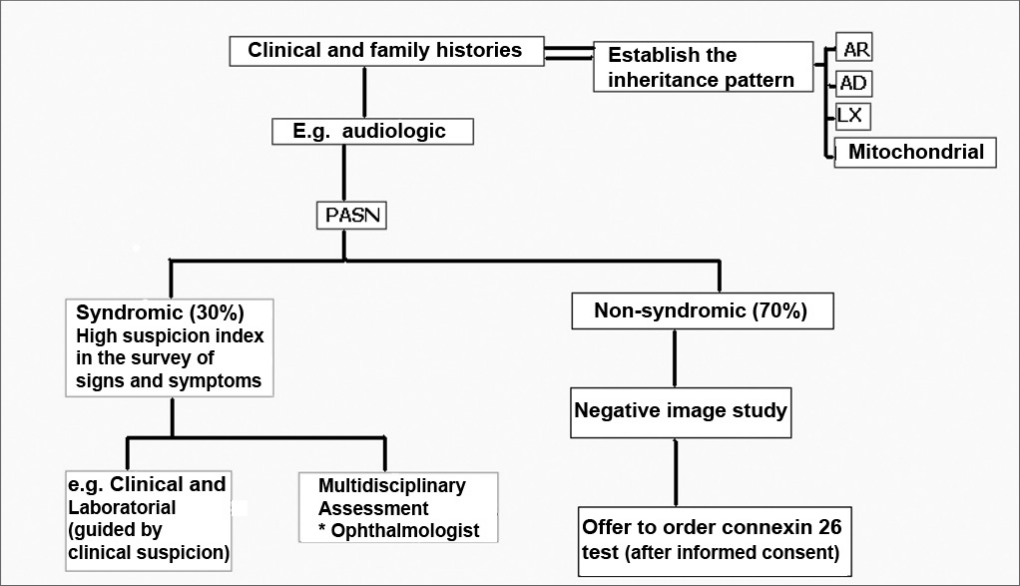
Genetic SNHL screening - The otorhinolaryngologist must have a high degree of suspicion in order to determine the syndromic forms (evidences of syncope or sudden death in other family members, in the syndromes of Jervell and Lange-Nielsen; enlargement of the vestibular aqueduct and positive perchlorate discharge test in the Pendred’s syndrome); the tests ordered must be guided by clinical suspicion; in more than 50% of the children with SNHL severe to profound, there are ophthalmologic alterations. When the SNHL happens alone and the image exams are negative, the Connexin 26 test should be proposed, respecting the patient’s autonomy and privacy. AR: recessive autosomal; AD: dominant autosomal; LX: X-linked; Mitochondrial: mitochondrial inheritance.

Genetic tests are part of the assessment and must help to confirm or rule out a specific diagnosis. The fast introduction of genetic tests in the clinical practice makes it necessary to search for information on the risks and benefits of such tests and also to what extent the exam introduction is useful and appropriate. GJB2 (connexin 26) is the most common cause of non-syndromic HL, therefore, it must the first step in mutation analysis investigation, after obtaining a signed informed consent. Peripheral blood and mouth mucosa cells collected with a swab may be analyzed. The methods employed in order to identify a GJB2 mutation are fast, relatively inexpensive, highly sensitive and specific, but limited, since the number of mutation points investigated is low. It may be useful in individuals with genetic hearing loss, of unknown etiology, without other clear clues and negative image studies (Figure 4).

**Figure 5:**
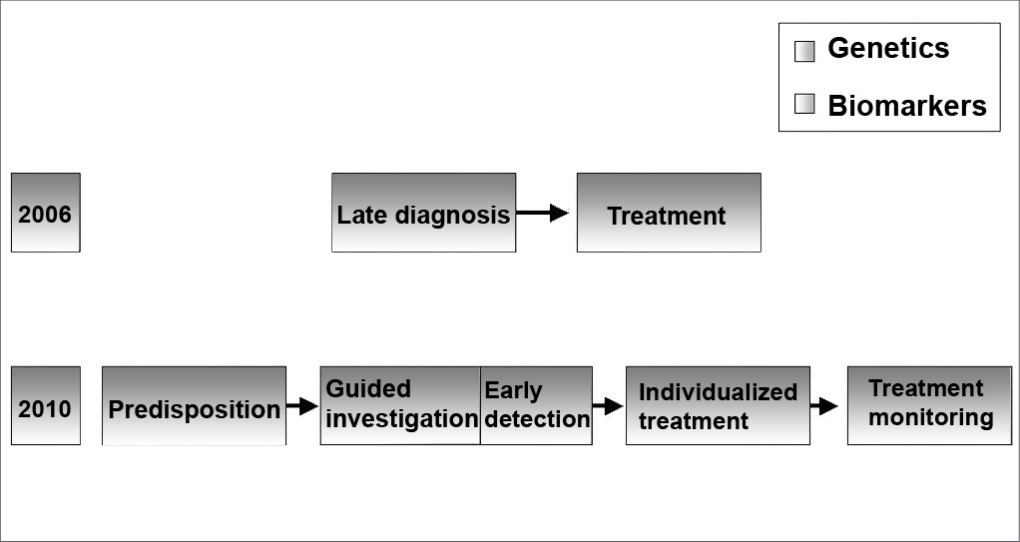
SNHL diagnosis - SNHL diagnosis will be driven by genetics and biomarkers. (Modified from Bell, 2004)

The diagnosis of SNHL, led by genetics and biomarkers will certainly progress in the next 10 years; early diagnosis, guided therapy and disease monitoring will replace the current late diagnosis and therapy paradigm^11^ (Figure 5).

## CONCLUSION

- Epidemiological data estimate that at least 50% of the pre-speech hearing losses are caused by genetic alterations.
- Clinical and family histories are extremely important in genetic SNHL diagnosis and contribute to determine inheritance patterns.
- Through high suspicion indexes, syndromic causes may be diagnosed or ruled out with careful evaluation, and the HL molecular basis may be better determined then before.
- Genetic tests and mitochondrial inheritance must be considered in families with multiple individuals affected, and the latter is ruled out if there is clear transmission from a man.
- In non-syndromic SNHLs, GJB^2^ mutation analysis must be proposed.
